# Primary Sjögren syndrome-associated acute interstitial nephritis and type 3 renal tubular acidosis in a patient with thin basement membrane nephropathy: A case report

**DOI:** 10.1097/MD.0000000000021644

**Published:** 2020-08-07

**Authors:** Tian Du, Xiaohang Liu, Wei Ye, Wenling Ye, Chao Li

**Affiliations:** a Department of Nephrology, Peking Union Medical College Hospital, Chinese Academy of Medical Sciences and Peking Union Medical College; b School of Medicine, Tsinghua University, No.1 Tsinghua Yuan; c Department of Cardiology, Peking Union Medical College Hospital, Chinese Academy of Medical Sciences and Peking Union Medical College, Beijing, China.

**Keywords:** primary Sjögren syndrome, renal involvement, acute tubulointerstitial nephritis, type 3 renal tubular acidosis, thin basement membrane nephropathy, case report

## Abstract

**Introduction:**

The kidney is one of the common extraglandular sites involved in primary Sjögren syndrome (pSS), with chronic tubulointerstitial nephritis (TIN) the most common pathology type. Renal involvement in pSS often presents as chronic TIN accompanied by type 1 or 2 renal tubular acidosis (RTA). Description of renal involvement as acute TIN with type III RTA in pSS has been rarely reported.

**Patient concerns:**

A 37-year-old woman was admitted with complaints of dry mouth, dry eyes, and progressive muscle weakness for 17 months. Two months before admission, the patient had a blood potassium level of 1.7 mmol/L.

**Diagnosis:**

Further tests confirmed pSS and type III RTA. Renal biopsy demonstrated acute TIN and thin basement membrane nephropathy (TBMN).

**Interventions:**

Full-dose corticosteroid (1 mg/kg/day) and cyclophosphamide (100 mg/day) were applied.

**Outcomes:**

The creatinine levels of the patient decreased 0.28 mg/dL (1.18–0.90 mg/dL) during 3-month follow-up.

**Conclusions:**

We reported a patient with pSS-associated kidney injury, presenting as acute TIN with type 3 RTA and TBMN. This case increases the awareness of a rare manifestation of pSS-associated kidney injury. In pSS-associated acute TIN, cyclophosphamide combined with full-dose corticosteroids may achieve good outcomes.

## 1 Introduction

Primary Sjögren syndrome (pSS) mainly affects salivary and lacrimal glands, but can also have extraglandular manifestations.^[1–5]^ Renal involvement is one of the common extraglandular manifestations in pSS, with a variable prevalence of 0.3% to 14% in all pSS patients, which is largely attributable to different pSS diagnostic criteria used and to the underestimation of tubular involvement in some studies.^[4–6]^ Seventy-five percent of renal involvement in pSS presents as tubulointerstitial nephritis (TIN) in pathology, of which the majority are chronic.^[1,4]^ Clinical presentations include type 1 renal tubular acidosis (RTA), type 2 RTA, renal concentrating defects, acquired Gitelman and Bartter syndromes, and nephritic syndrome.^[4,5]^ Descriptions of kidney involvement as acute TIN with type 3 RTA have been much rarer.

In this report, we described a rare case of pSS-associated acute interstitial nephritis, presenting as type 3 RTA. In addition to interstitial changes, there was thin basement membrane nephropathy (TBMN) identified by electron microscope. Her renal function was improved when treated with cyclophosphamide (CTX) and high-dose corticosteroids combination.

## 2 Case Presentation

**Figure 1:**
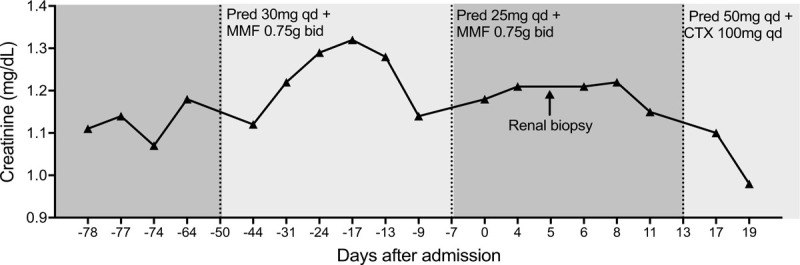
The trend graph of creatinine before and after admission (day 0). Prednisone was adjusted to 25 mg qd at day −7 as creatinine significantly dropped from day −17 to day −9. CTX = cyclophosphamide, MMF = mycophenolate mofetil, Pred = prednisone

A 37-year-old woman suffered from dry mouth and dry eyes since June 2017. In July 2018, she experienced progressive muscle weakness, which finally evolved into an episode of paralysis. The patient was admitted to our hospital in November 2018. Her baseline serum creatinine level was normal in June 2018. Two months before admission, the patient had a blood potassium level of 1.7 mmol/L, sodium of 144 mmol/L, and chloride of 109.5 mmol/L. The potential of hydrogen (pH) was 7.36 and the concentration of bicarbonate was 20.7 mmol/L in the blood gas analysis. The anion gap was within the normal range (13.8 mmol/L). Her serum creatinine was 1.17 mg/dL, and the antinuclear antibody was 1:320 positive. Anti-SSA and anti-SSB antibodies were negative. Urine analysis showed a pH of 7.50, glucose and protein were negative in the urine, and hematuria was not detected. The 24-hour urine protein was 0.69 g. Meanwhile, urine β2 microglobulin (0.64 mg/L), α1 microglobulin (12.1 mg/L) and 24-hour urine potassium (98.7 mmol) were elevated. Postural stimulation for excluding primary aldosteronemia was negative. The Schirmer test and fluorescein staining were normal. Her unstimulated salivary flow rate was <0.1 mL/minute. Focal lymphocytic sialadenitis was confirmed by labial gland biopsy. The patient was diagnosed as pSS and RTA at local hospital, and thus was administrated with 30-mg prednisone daily and 750-mg mycophenolate mofetil (MMF) twice a day. Totally 2 to 6 g potassium was supplemented daily. Accordingly, the patient's paralysis was relieved. Routine tests showed potassium of 2.8 to 3.4 mmol/L, whereas no significant improvement was achieved with creatinine levels of 1.12-1.32 mg/dL (Fig. 1).

**Figure 2:**
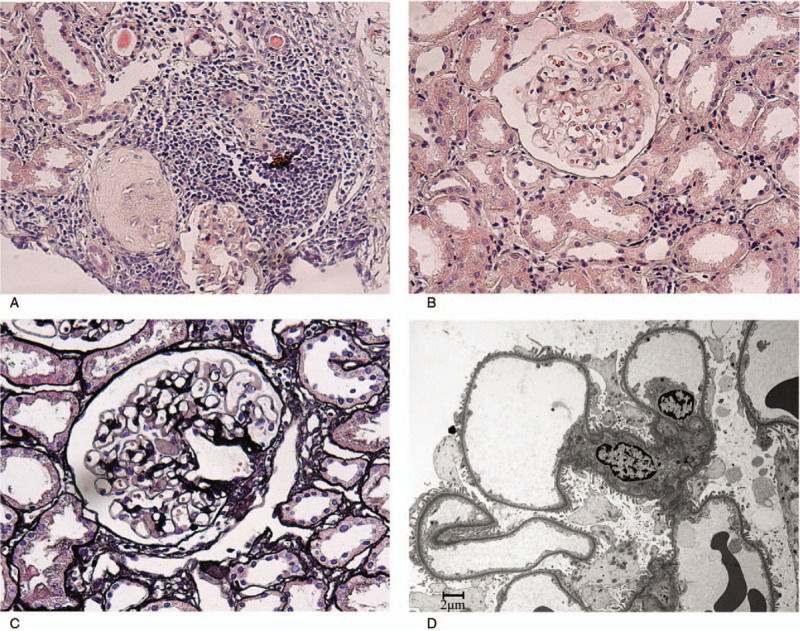
Renal biopsy findings of the case. (A) Multifocal interstitial inflammation with lymphocytes, and plasma cells (hematoxylin-eosin stain, ×100). (B) No hypercellularity in glomeruli (hematoxylin-eosin stain, ×200). (C) No GBM thickening in glomeruli (Jones silver stain, ×200). (D) Electroscopic imaging showed diffusely thin glomerular basement membrane with thickness of 180 to 240 nm. Electron dense deposits were not observed (×4000).

On admission, laboratory tests showed blood potassium of 3.0 mmol/L, creatinine of 1.18 mg/dL, and 24-hour urine protein of 0.14 g (for more details, refer to Table 1). Fractional bicarbonate excretion was 6.6%. Type 3 RTA was diagnosed, which indicated tubulointerstitial changes. Considering the patient had pSS, pSS-associated TIN was highly suspected. To further specify the cause of the kidney abnormality, and guide future treatment, renal biopsy was performed. Renal pathology (Fig. 2) was remarkable for the diffuse and mild fibrosis and evident infiltration of inflammatory cells in the renal interstitium on light microscopy. Glomeruli were intact. Focal and segmental IgG deposits (+) were observed in the glomerular mesangial area and capillary loops. Electron microscopy revealed diffusely thin glomerular basement membrane and infiltration of lymphocytes and monocytes in the interstitium (Fig. 2).

**Table 1:**
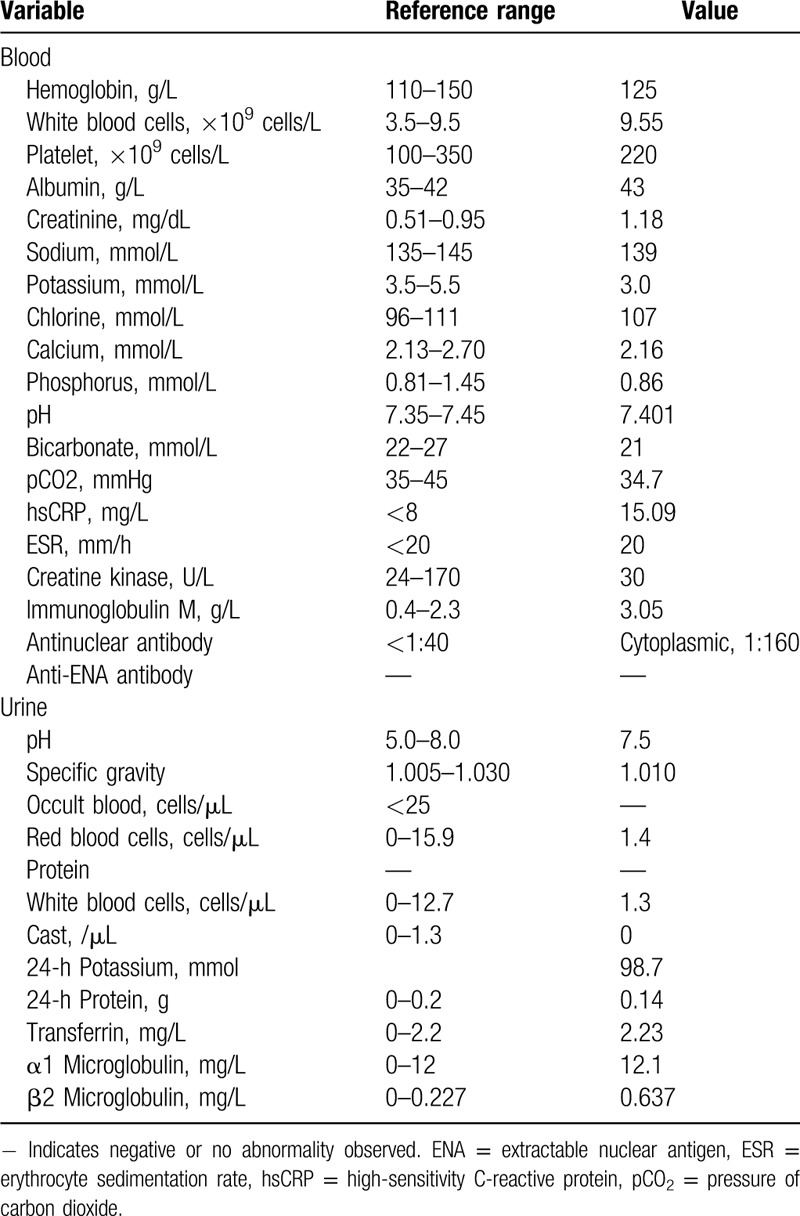
Laboratory data on admission.

Therefore, acute kidney injury, acute interstitial nephritis, type 3 RTA caused by pSS were diagnosed. Oral prednisone 50 mg once a day (1 mg/kg/day) and CTX 100 mg once a day were given. Prednisone was tapered 5 mg/week after 1 month. At the same time, 3 to 6 g potassium chloride was supplemented every day. During the 3-month follow-up, the blood potassium level maintained in the normal range (3.4–3.6 mmol/L) and the serum creatinine decreased (0.90–1.02 mg/dL).

## 3 Discussion

The majority of kidney involvement in pSS patients is chronic TIN, and usually presents as type 1 or type 1 RTA.^[4,5]^ To the best of our knowledge, we reported the first case of pSS-associated acute TIN with type 3 RTA. In addition to interstitial changes, the patient also had TBMN.

First, the patient presented as renal losses of potassium at the onset of the disease. RTA was identified with further examinations. Fractional exertion of bicarbonate was used to determine different types of RTA, with fractional exertion >15% for type 1 RTA, and fractional exertion <5% for type 2 RTA.^[7]^ This patient had a fractional exertion of 6.6%, implying type 3 RTA, which had features of both type 1 and 2 RTA. As far as we know, this was the first reported case of pSS-associated type 3 RTA.

Secondly, the diagnosis of RTA indicated the involvement of tubulointerstitial in this patient. pSS tubulointerstitial injury often presents as chronic interstitial nephritis in kidney biopsy findings.^[1,8]^ Maripuri et al^[1]^ reported that among 17 pSS cases with TIN, 11 (65%) cases were chronic TIN, 6 (35%) cases were acute TIN. Similar results were reported by Ren et al^[8]^ in 41 Chinese pSS patients with renal involvement, of which 83% (n = 33) were chronic TIN. The precise diagnosis of TIN is verified by renal pathology. Kidney biopsy helps specify chronic and acute TIN, excludes causes of tubulointerstitial injury (eg, light chain deposition disease), and also guides further treatment.^[9,10]^ The histologic analysis of this patient demonstrated acute TIN, which was relatively uncommon in pSS patients.

There is no consensus treatment for extraglandular manifestations in pSS patients. Treatment options include corticosteroids, calcineurin inhibitor, CTX, and MMF.^[11]^ Corticosteroids are fundamental in the treatment for pSS-associated kidney injury. Corticosteroid (≥0.5 mg/kg/day) without other immunosuppressors was reported to improve the eGFR of patients with pSS-associated interstitial injury.^[5]^ Only a few groups studied the effect of the combination of corticosteroids and immunosuppressive agents in the treatment of pSS-associated renal interstitial injury. Maripuri et al^[1]^ treated 2 pSS patients with renal interstitial injury with prednisone (30–60 mg/day) and CTX. The 2 patients had stable eGFR in 5-year follow-up. Shen et al^[12]^ divided 70 pSS patients with chronic TIN into the corticosteroids (≥15 mg/day) and CTX (0.6–0.8 g/month) combination group (n = 14) and corticosteroids (≥15 mg/day) alone group (n = 56). After 12-month follow-up, the group with combination treatment had better eGFR improvement (21.35 ± 19.63 vs 2.72 ± 19.11 mL/min/1.73m^2^, *P* = .006). Evans et al reported the use of T, B cell inhibitor—MMF on 11 pSS patients with TIN, and found MMF (1000–1500 mg/day) combined with prednisolone (5–20 mg/day) improved eGFR of those patients. Currently, there are no large, randomized clinical trials directly comparing the effect of MMF and CTX on pSS patients with TIN. The best doses of them also need to be further investigated. The patient in our report was initially treated with moderate-dose corticosteroid (0.5 mg/kg/day) combined with MMF, whereas the level of creatinine did not improve significantly. A kidney biopsy was performed for treatment decisions. Because the pathology of kidney biopsy mainly presented acute kidney injury changes, we treated the patient with high-dose corticosteroids and CTX. Significant remission of creatinine occurred with a 3-month treatment, which served as an effective practice for the use of CTX combined with high-dose corticosteroid in the treatment of pSS patients with acute TIN. We propose that future research should be undertaken to determine the best treatment for pSS associated TIN, especially for acute TIN unresponsive to medium-dose corticosteroids.

Lastly, electron microscopy demonstrated concurrent TBMN. Yang et al^[13]^ also reported a case having both pSS-associated kidney injury and TBMN, whereas whether the concurrence of the 2 diseases has underlying association is still unknown.

## 4 Conclusions

In summary, pSS-associated kidney injury could present as a rare combination of type 3 RTA and acute interstitial nephritis. For pSS-associated acute interstitial nephritis, we suggest high-dose corticosteroids and CTX combination in treatment, especially when the patient is unresponsive to medium-dose corticosteroids.

## Author contributions

**Conceptualization:** Tian Du, Xiaohang Liu, Chao Li.

**Data curation:** Tian Du, Xiaohang Liu, Chao Li.

**Investigation:** Tian Du, Xiaohang Liu, Wei Ye, Wenling Ye, Chao Li.

**Supervision:** Chao Li.

**Visualization:** Tian Du.

**Writing – original draft:** Tian Du, Xiaohang Liu.

**Writing – review & editing:** Wei Ye, Wenling Ye, Chao Li.
